# Prevalence of intimidation, harassment, and discrimination among resident physicians: a systematic review and meta-analysis

**DOI:** 10.36834/cmej.57019

**Published:** 2020-03-16

**Authors:** Anees Bahji, Josephine Altomare

**Affiliations:** 1 Department of Psychiatry, Queen’s University, Ontario, Canada; 2 Department of Public Health Sciences, Queen’s University, Ontario, Canada

## Abstract

**Background:**

The aim of this study was to establish the prevalence of intimidation, harassment, and discrimination (IHD) reported by resident physicians during their training, to identify factors associated with reported IHD, and to identify adverse sequalae associated with IHD.

**Methods:**

This review followed the PRISMA guidelines. Eight electronic databases were searched for cross-sectional studies reporting the prevalence of IHD among resident physicians. Prevalence estimates were pooledacross studies using random-effects meta-analysis, with variance stabilization using Tukey double arcsine transformation. Heterogeneity was assessed with forest plots, the *I^2^* statistic, subgroup analyses, and multivariate meta-regression.

**Results:**

52 cross-sectional studies were included in the meta-analysis. The overall pooled prevalence of IHD was 64.1% (95% confidence interval [CI], 51.0-77.1). Verbal, physical, and sexual IHD were the most common forms of IHD reported by residents. Training status (55.5%), gender (41.7%), and ethnicity (20.6%) were the most commonly cited risk factors for IHD. The most common sources of IHD were relatives/friends of patients, nurses, and patients (cited by 50.7%, 47.8, and 41.7%, respectively).

**Conclusions:**

The prevalence of IHD among resident physicians is high and associated with multiple negative outcomes, including burnout. Despite the availability of multiple anti-IHD interventions, reports of IHD appear to be rising in many residency programs.

## Abstract (fr)

**Objectif:**

Cette étude visait à établir la prévalence de l’intimidation, du harcèlement et de la discrimination (IHD) signalés par les médecins résidents pendant leur formation, àdéterminer les facteurs associés au signalement del’IHDet à identifier les effets néfastes associés à l’IHD.

**Méthodes:**

Cette revue a suivi les lignes directricesde la méthode PRISMA surles revuessystématiques et les méta-analyses. Nous avons utiliséhuit bases de données électroniques pour trouver des études transversales ayant fait état de la fréquence des comportements d’IDH parmi les médecins résidents. Les estimations sur la prévalencede l’IHD ont été obtenues sur la basede méta-analyses d’effets randomisés, avec une la stabilisation de la variance grâce à la double transformation arcsine de Tukey. L’hétérogénéité a été évaluée qualitativement à l’aide de graphiques en forêt et quantitativement en utilisant la statistique I *^2^*, des analyses de sous-groupes et la méta-régression multivariée.

**Résultats:**

52 études transversales ont répondu aux critères d’inclusions. L’ensemble des prévalences regroupéesd’IHD était de 64,1 % (95 % IC, 51.0-77.1). Les formes verbales, physiques et sexuelles étaient les IHD les plus fréquemment signalées par les résidents. Le niveau de formation (55,5 %), le sexe (41,7 %) et l’ethnicité (20,6 %) étaient les trois facteurs de risque les plus couramment cités concernant l’IHD.. Les sources les plus communes de l’IHD étaient les parents/amis des patients, les infirmières et les patients (cité par 50,7 %, 47,8 % et 41,7 %, respectivement).

**Conclusions:**

La prévalencede l’IDH auprèsdes médecins résidents est élevée et associée à plusieurs conséquences négatives, notamment l’épuisement professionnel. Malgré la disponibilitéde plusieurs interventions anti-IHD, les signalements d’IDH semblent être à la hausse dans plusieurs juridictions.

## Background

The Canadian human rights commission defines intimidation, harassment, and discrimination (IHD) as unwanted physical or verbal behaviours that are offensive or humiliating, which can occur on the basis of race, religion, sex, age, disability, or other grounds.^1^Specifically, intimidation refers to the use of authority to inappropriately influence behaviour.^2^ Harassment is defined as unwelcome or vexatious conduct that occurs on the basis of the perceived status of the target, be it ethnicity, gender, sexual orientation, age status, or other attributes.^3^^,^^4^ Discrimination denotes to the unjust or prejudicial treatment of different categories of people.^5^ In learning environments, IHD often induces fear or anxiety in the learner, causing generally detrimental effects on the learner’s ability to succeed.^6^

In recent years, the deleterious impacts of IHD on medical trainees acrossall stages of training have been recognized internationally.^7^ The psychiatric sequelae of exposure to IHD have been particularly well studied, with a recent systematic review finding that IHD increased the risk anxiety disorders, sleep disorders, eating disorders, posttraumatic stress disorder, and suicide attempts by three to 16-fold – regardless of sex or age.^8^Therefore, IHD appears to be associated with an increased prevalence of psychopathology as well as specific patterns ofpsychopathology.^9^^,^^10^

IHD also has negative impacts on learning and educational outcomes among medical trainees, with recent studies showing that trainees are less likely to pursue a medical speciality that they perceive to be particularly hostile.^2^ Exposure to IHD during training also influences a trainee’s academic trajectory because it affects their ability to communicate, concentrate, and collaborate.^11^^,^^12^

Medical students and resident physicians are especially vulnerable to IHD for a number of reasons.^13^ As trainees are learners who are dependent on their supervisors and senior residents for promotion through the medical education system, this creates a power gradient, whichsets the stage for IHD.^14^ In medicine, IHD behaviours are often rationalized as ‘rites of passage’ or ‘beneficial to training’^15^—not unlike the role of distorted cognition in initiating and maintaining cycles of sexual abuse and assault.^16^Similarly, a number of cultural factors within medicine that discourage reporting of IHD and encourage victimization have contributed tothe phenomenon of “whistleblowing” within medicine.^14^^,^^17^^-^^21^

In response to mounting criticism,^14^^,^^18^^,^^22^^-^^24^ multiple professional organizations, including the Royal College of Physicians and Surgeons of Canada, the Canadian Medical Association, Resident Doctors of Canada, the American Medical Association, and the World Medical Association, have published position papers denouncingIHD in medicine.^13^^,^^25^^-^^29^ Several Canadian medical schools, including Queen’s University, Memorial University of Newfoundland, and the University of Manitoba, have developed IHD policy statements and imposed IHD reporting protocols.^30^^-^^32^ These documents provide examples and definitions of IHD, outline informal and formal resolution processes, while emphasizing confidentiality, fairness, and transparency.^10^^,^^30^^,^^33^

Despite these efforts, there is evidence that IHD continues, and may even be on the rise within medicine.^6^ National surveys conducted by Resident Doctors of Canada in 2012 and 2018 identified that 73% and 78.2% of Canadian residents, respectively, reported at least one instance of IHD during residency, showing a 5% increase over this six-year span.^34^^,^^35^ While these estimates may also reflect decreased barriers to reporting, particularly as many surveys are kept anonymous, the effectiveness of anti-IHD infrastructure is unclear.^14^^,^^18^^,^^22^^-^^24^

Previous reviews have described IHD across different Canadian medical education settings. In 2014, Karim and Duchererreported that between 45% and 93% of residents reported at least one instance of IHD during residency.^6^^,^^36^ In another study, two Canadian family medicine programs reported that 33.7% of trainees had experienced IHD during residency.^2^In another survey, 36% of neurosurgery residents reported IHD during their training.^37^Internationally, nearly 50% of surveyed medical students, residents, and fellows report experiencing or witnessing at least one form of IHD during their training in the United Kingdom, United States, Japan, Nigeria, and Canada.^6^^,^^12^^,^^36^^,^^38^

A prior, comprehensive systematic review conducted in 2014 by Fnais and colleagues^36^found that the pooled prevalence for IHD among any medical trainee was 59.4% (n = 51 studies, 38,353 trainees, 95% confidence interval [CI]: 52.0%-66.7%). Among residents, the authors’ estimate of IHD was 63.4% (n = 19 studies, 11,193 residents, 95% CI: 53.6%-73.2%). The authors reported that residents cited gender discrimination as the most common form of abuse (n = 3 studies, 1,315 residents, prevalence: 66.6%, 95% CI: 58.7%-74.5%), followed by verbal harassment (n = 12 studies, 9,867 residents, prevalence: 58.2%, 95% CI: 45.5%-70.9%). Among residents, the least common form of IHDwas racial discrimination (n = 3 studies, 3,261 trainees, prevalence: 26.3%, 95% CI: 24.2%-28.3%). Heterogeneity was significant across these studies.

Allied health professionals, including nurses,^39^^,^^40^ physician assistants,^41^ home health aides,^42^ and social workers,^43^ have also experienced high rates of IHD. Converging sources of data suggest that IHD is not limited to North America or to medical trainees specifically, but may represent a larger, and more systemic issue that affects medicine on the whole.

Several factors related to IHD research make it an intrinsically difficult topic to study by way of meta-analysis. The overreliance on cross-sectional surveys (prone to recall bias) and the subjective nature of IHD terminology (often utilizing overlapping definitions) increase heterogeneity between studies, limiting the extent to which results can be pooled. Still, several previous reviews have pooled the available IHD literature^2^^,^^6^^,^^8^^,^^33^^,^^36^^,^^38^by collapsing multiple forms of IHD, several groups of trainees, and countries or regions, often due to the limited number of studies identified by any individual review. While the review done by Fnais and colleagues^36^is the most comprehensive to date, there are opportunities to expand their findings by identifying more resident-specific studies.This may in turn enable additional subgroup analyses, greater description of between-study heterogeneity, andan improved understanding of the contextual factors involved in IHD among residents.

Therefore, the objective of the study was to establish the prevalence of IHD reported by resident physicians during their training, to identify factors associated with reported IHD, and to identify adverse sequalae associated with IHD.

## Methods

### Ethics

Research ethics board approval and consent procedures were waived as this study was a meta-analysis of publicly available studies.

### Search strategy

A systematic review protocol was developed with the support of an experienced research librarian using the Preferred Reporting Items for Systematic Reviews and Meta-Analyses (PRISMA) guidelines.^44^ Medical Subject Headings (MeSH) and free-text searches related to IHDamongresident physicians were used to search the following seven electronic databases: PubMed, MEDLINE, EMBASE, PsycINFO, CINAHL, Allied and Complementary Medicine (AMED), and the Cochrane Library. The following MeSH terms were used when searching MEDLINE: “Internship and Residency,” “Resident,” “Medical Residency,” “Intimidation and Harassment.” We searched each database from inception to August 1, 2018, with an updated search conducted on May 28, 2019. Specific definitions and examples of IHD are described in Appendix A, whilethe detailed search strategies for each database are described in Appendix B. The reference lists of included studies and reviews were scanned for additional articles. The ProQuest database of dissertations and theses was also searched for relevant grey literature to supplement findings from published studies.^45^^,^^46^

### Eligibility criteria

English-language studies reporting the prevalence of self-reported IHD among resident physicians—or those where the prevalence could be computed using raw data reported by the studies—were eligible for inclusion in this review. If studies reported data on other groups (such as staff physicians, medical students, or allied health practitioners), resident-specific data were extracted. No restrictions were placed on geographic location, stage of training, type of residency program, date of dissemination, or subtype of IHD.

### Outcome measures

Outcome measures were defined *a priori* for consistency with previous reviews of IHD.^6^^,^^36^The primary outcome was the prevalence of IHD. Secondary outcomes included:

- Sources of IHD (staff physicians, residents, medical students, patients, relatives of patients, nurses, and other staff)
- Risk factors for IHD (gender, training status, sexual orientation, ethnicity, culture, language, and other factors)
- Reporting of IHD (awareness, reporting rates, perceived barriers to reporting)
- Impacts of IHD (general satisfaction, quality of life, self-rated mental health, mental health screening)
- Proposed resources or solutions to IHD (education, training, policies, infrastructure, supports, wellness, access to an ombudsperson, access to a physician or counsellor, and career or other forms of advice)

Crude prevalence estimates were determined by dividing the total number of residents reporting IHD—overall or by subtype—by the total number of survey respondents. For example, if 10 from a total of 50 survey respondents reported sexual harassment, the prevalence of sexual harassment from that particular study was calculated as 20%.

### Selection of studies

Both authors screened all articles for inclusion using a two-stage process, supported by Rayyan, a web-based systematic review software.^47^ During the first stage, articles were excluded on the basis of title and abstract. Articles deemed relevant by either author progressed to the second stage, where full text versions of all articles were screened againstthe eligibility criteria. All disagreements were resolved by consensus.

### Data extraction and management

A data collection sheet was developed in Microsoft Excel; study coding variables are described in Appendix C. Both authors independently collected data, and discrepancies were resolved by consensus. If there were multiple companion publications reporting on data from the same population, only the most recent analysis was considered. Across studies, prevalence was usually reported as percentages or proportions. Missing data were not included.

### Assessment of risk of bias in included studies

To assess study quality, the Risk of Bias tool (RoBT) for prevalence studies developed by Hoy and colleagues^48^was used because of its use in other meta-analyses of prevalence studies,^49^^-^^52^its high interrater reliability,^53^^,^^54^ previous validation,^55^^,^^56^ and simplicity.^55^^,^^56^ ThisRoBT consists of ten items addressing four domains of bias and an eleventh summary risk of bias item (described in Appendices F and G). The four domains of bias assess external and internal validity using forced choice responses (yes/no). Each “yes” received a score of 1, while"no” responses received a scored of 0: the total possible score was ten. “Low” risk of bias was defined as scoring 0 to 2 points, “moderate” was defined as 3 or 4 points, while “high” was defined as 5 or more points.^49^^-^^52^ Both authors independently scored studies using the RoBT; all disagreements were resolved by consensus. Inter-rater agreement was quantified with the kappa coefficient.^57^Kappa was 0.83, indicating moderate-high agreement across raters.

### Statistical methods

All analyses were conducted using the Open Meta Analyst.^58^IHD prevalence estimates were pooled usinga random-effects model,generating an overall prevalence and accompanying 95% confidence intervals [CIs]. Statistical heterogeneity was assessed using tau^2^, *Q*, and *I^2^*and with forest plots.^59^ To stabilize variance across proportions and percentages, the arcsine transformation was applied, which allowed the sampling distribution to better approximate a normal distribution.^60^^-^^62^Leave-one-out meta-analysis was applied as a method of sensitivity testing to measure the robustness of the results.^63^^-^^65^

## Results

### Results of the search

**Figure 1:**
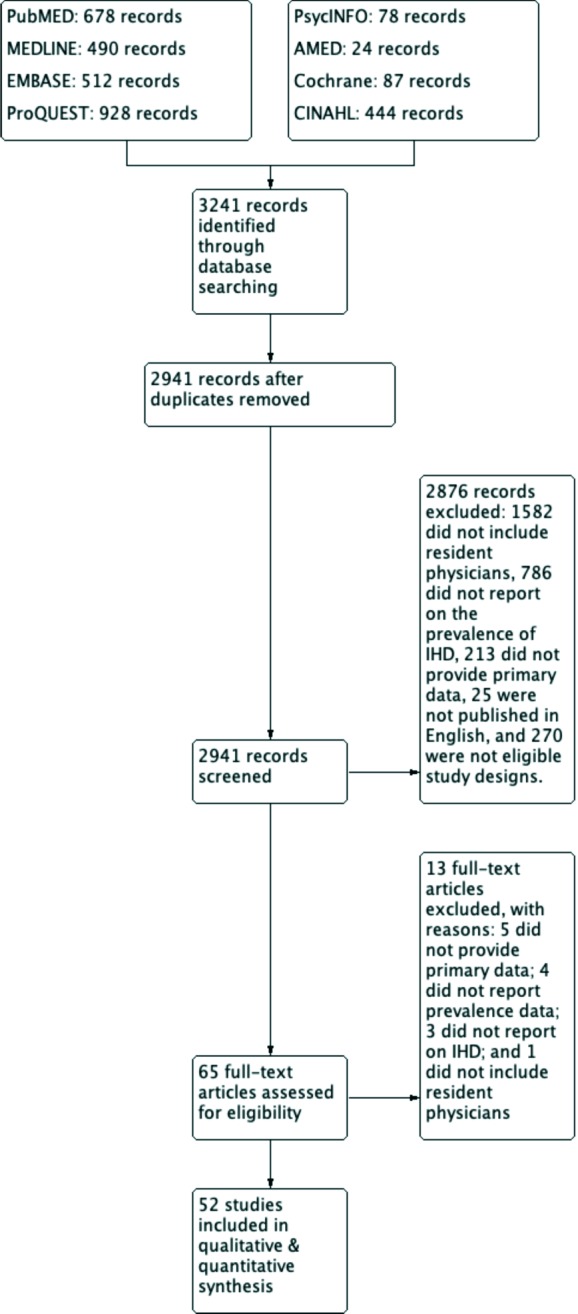
PRISMA study flow diagram

The literature search yielded 2,941 unique citations (Figure 1). From these, 2,876 were excluded because they did not include resident physicians (n = 1,582), did not report IHD (n = 786), did not provide primary data (n = 213), were not published in English (n = 25), or were not eligible study designs (n = 270). The remaining 65 records were obtained andreviewed in full. Reasons for exclusion at the full-text review stage included that the study did not provide primary data (n = 5), did not report IHD (n = 3), did not report prevalence or risk factors of IHD (n = 4), or did not include resident physicians (n = 1). A total of 52 cross-sectional studies fulfilled the inclusion criteria for this meta-analysis.

### Study and resident physician characteristics

Table 1 describes study and participant characteristics.Most studies were conducted in the United States (44%, n = 23), Canada (21%, n = 11), or the United Kingdom (13%, n = 7).

**Table 1:** Study characteristics

Study	Year	Country	Survey	Source	Target
**Acik et al**.	2008	Turkey	Mailed	First University Medical University	All Turkish medical schools
**Al-Shafaee et al**.	2013	Oman	Electronic	Sultan Qaboos University	All training centres in the Oman Medical Specialty Board
**Alimohammadi et al**.	2013	Iran	In-person	ShahidBesheshti University Medical School	Central hospitals in Tehran, Mashhad, Ahwax, and Tabriz
**Baldwin et al**.	1996	USA	Mailed	Rush Primary Care Institute	Senior residents at 10 regionally distributed US medical schools
**Baldwin et al**.	1997	USA	Mailed	American Medical Association (AMA)	Senior residents at 10 regionally distributed US medical schools
**Baldwin et al**.	1994	USA	Mailed	Rush Medical College	All 2^nd^ residents in the AMA National Database (10% random sample)
**Barlow & Rizzo**	1997	USA	Mailed	Wright Patterson Air Force Base	Cohort of surgical residents from the AMA National Databank
**Behnam et al**.	2011	USA	Electronic	West Virginia University	Cohort of emergency residents from the AMA National Database
**Black et al**.	1994	USA	In-person	Washington University School of Medicine	Child psychiatry residents at three training hospitals
**Carr et al**.	1991	Canada	Mailed	University of Toronto	All Canadian residency programs outside of Quebec
**Chadaga et al**.	2016	USA	Electronic	Advocate Health Care	National sample of residents and fellows
**Chaimowitz&Moscovitch**	1991	Canada	Mailed	McMaster University	All Canadian residency programs outside of Quebec
**Cohen & Patten**	2005	Canada	Electronic	Universities of Calgary & Alberta	All Members of Professional Association of Residents of Alberta
**Cohen et al**.	2008	Canada	Electronic	Resident Doctors of Canada	All Canadian residency programs outside of Quebec
**Cook et al**.	1996	Canada	Mailed	McMaster University	Residents in 7 Training Programs at McMaster University
**Crutcher et al**.	2011	Canada	Mixed	Universities of Calgary and Alberta	All family medicine graduates from the two universities
**Daughterty et al**.	1998	USA	Mailed	American Medical Association	All 2^nd^ residents in the AMA National Database (10% random sample)
**Deringer &Caligor**	2014	USA	Electronic	New York University School of Medicine	All psychiatry resident at New York University School of Medicine
**Dvir et al**.	2001	USA	Electronic	University of Massachusetts Medical School	All programs enrolled in the APA Leadership Fellowship
**Fink et al**.	1991	USA	Mailed	Institute of Pennsylvania Hospital	11 residency training programs in Pennsylvania
**Finucane & O'Dowd**	2005	Ireland	Mailed	Medical Council of Ireland	All interns with Irish Addresses in the Irish Medical Council Database
**Fnais et al**.	2013	Saudi Arabia	In-person	King Saudi University College of Medicine	National Guard Hospitals in Riyadh, Jeddah, and Al-Ahsa’a
**Gray**	1989	USA	Mailed	University of Southern California	All psychiatric trainees at a county hospital
**Hoosen &Callghan**	2004	UK	Mailed	Penn Hospital	All Psychiatric Trainees in the West Midlands
**Hostiuc et al**.	2014	Romania	Electronic	Carol Davila University of Medicine	Residents across all specialties doing their bioethics module/rotation
**Judy &Veselik**	2009	USA	Electronic	Loyola University Medical Centre	Residents at all training levels from 25 pediatric programmes
**Keeley et al**.	2005	UK	Mailed	Glasgow Royal Infirmary	All junior residents in National Health Service trusts
**Komaromy et al**.	1993	USA	Mailed	University of California, San Francisco	All internal medicine residents at San Francisco General Hospital
**Kozlowska et al**.	1997	Australia	Mailed	Noval North Shore Hospital	All New South Wales Trainees
**Li et al**.	2010	USA	Mailed	Jacobi Medical Center	Sample of 10 EM Residency Programs in New York City
**Mackin**	2001	UK	Telephone	St. Mary’s Hospital	75 pediatric trainees across 3 regions in the UK
**McNamara et al**.	1995	USA	Mailed	Medical College of Pennsylvania	American Board of Emergency Medicine
**Milstein**	1987	USA	Mailed	Indiana University School of Medicine	All enrolled internal medicine residents
**Milstein et al**.	1987	USA	Mailed	Indiana University School of Medicine	All psychiatry residents at Indiana University School of Medicine
**Morgan & Porter**	1999	UK	Mailed	St. George’s Hospital Medical School	Random Sample of all psychiatric trainees across all NHS Trusts
**Nagata-Kobayashi et al**.	2009	Japan	In-person	International Medical Center of Japan	All trainee physicians at 37 Japanese Hospitals
**Ogunsemi et al**.	2010	Nigeria	In-person	Olabisi Onabanjo University Hospital	Association of Resident Doctors of the Nigerian Teaching Hospital
**Paice& Smith**	2009	UK	Electronic	Postgraduate Medical Education & Training	All of the trainee doctors in national educationally-approved posts
**Paice et al**.	2004	UK	Electronic	London Deanery	Doctors in training in London North of the Thames
**Pieters et al**.	2005	Belgium	Mailed	Flemish Training Committee for Psychiatry	Random sample of all psychiatric trainees from Dutch-speaking Belgium
**Quine**	2002	UK	Mailed	University of Kent at Canterbury	1000 Trainee Physicians enrolled in the British Medical Association
**Resident Doctors of Canada**	2011	Canada	Electronic	Residents Doctors of Canada (RDoC)	All Canadian residency programs outside of Quebec
**RDoC**	2012	Canada	Electronic	RDoC	All Canadian residency programs outside of Quebec
**RDoC**	2013	Canada	Electronic	RDoC	All Canadian residency programs outside of Quebec
**RDoC**	2018	Canada	Electronic	RDoC	All Canadian residency programs outside of Quebec
**Recupero et al**.	2005	USA	Mailed	Brown University Medical School	All medicine residents at four affiliated teaching hospitals
**Ruben et al**.	1980	USA	In-person	University of Southern California	All psychiatry residents at the University of Southern California
**Schnapp et al**.	2016	USA	In-person	Mount Sinai Hospital Ichan School of Medicine	All emergency residents training in New York City
**Schwartz & Park**	1999	USA	Mailed	State University of New York Health Science	Random sample of all psychiatric trainees in accredited AMA programs
**Vaninevald et al**.	1996	Canada	Mixed	McMaster University	13 of 16 Canadian Internal Medicine Programs
**Vukovic et al**.	1996	USA	Mailed	Family Health Care of Wadsworth	All female family practice trainees in the AMA Database
**Walter et al**.	2003	Australia	Mailed	Central Sydney Area Health Service	All nationally registered psychiatric trainees

All studies were conducted between 1980 and 2018, and the number of residents per study ranged from 31 to 50,240 (see Appendix D). Only seven studies were conducted in-person, while the remaining 45 studies were conducted by mail or electronically; however, all studies obtained data directly from respondent.

The population of residents varied substantially across the included studies (see Appendix D). A total of 63,378 respondents were included across all studies (48% female). The overall rate of response was 51% (63,378/125,343), while the mean response rate per study was 64% (standard deviation [SD], 22%). Respondents were distributed across all postgraduate training levels (24% in postgraduate year 1 [PGY1], 47% in PGY2, 23% in PGY3, 5% in PGY4, and 1% in PGY5 or higher). 37% of studies (n = 19) surveyed all specialties, 29% (n = 15) focused on Psychiatry only, 8% (n = 4) surveyed Emergency Medicine residents exclusively, and 4% (n = 2) studied Internal Medicine trainees and Family Medicine trainees, respectively.

### Types and sources of IHD among resident physicians

The types of IHD reported by studies included physical (73%), verbal (63%), sexual (52%), work-as-punishment (19%), academic (17%), and revocation of privileges (12%). Forty-four studies (85%) reported on at least one source of IHD (range: 1–7). In order of decreasing frequency, the most common sources were relatives/friends of patients (cited by 50.7% of respondents), nurses (47.8%), patients (41.7%), consultants/attending physicians (39.0%), other residents (35.8%), medical students (10.6%), and other staff (9.5%).

### Risk factors for IHD among resident physicians

Sixteen studies (32%) reported one or more risk factors for IHD (range: 1-6). In order of decreasing frequency, the most common risk factors were training status (cited by 55.5% of respondents), gender (41.7%), ethnicity (20.6%), culture (9.9%), sexual orientation (2.5%), and language (2.3%).

### Methodological quality

Among the 52 studies, 37 (71%) recruited a nationally representative sample population. However, only ten (19%) used a random sample to obtain a truly representative sample of the average resident physician. Eighteen studies (35%) used a validated survey instrument. All but two studies used the same mode of data collection for all study respondents.^66^^,^^67^ Forty-seven studies (90%) provided definitions and example of IHD for respondents; the remaining five studies intentionally excluded IHD definitions to promote completion of the survey without restraint. Forty-one studies (79%) had a survey response rate greater than 50%.

The majority of studies did not provide full demographic descriptions of their resident populations.For example, 13 (25%) studies did not report the sex distribution of respondents, 29 (56%) did not report the whereabouts of the residents’ training, 20 (39%) did not report the residents’ year of training, and 37 (71%) did not report the residents’age distribution. None of the studies controlled for age or gender to improve the comparability of results across studies.

### Meta-analysis of IHD prevalence among resident physicians

**Figure 2:**
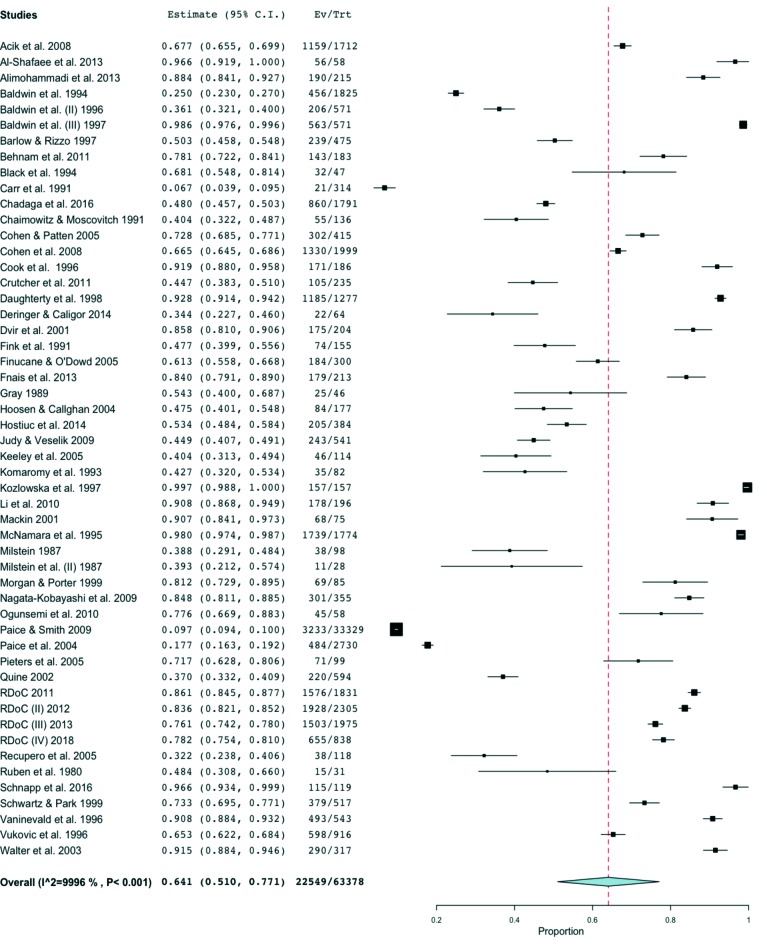
Meta-analysis of prevalence of overall Intimidation, Harassment, and Discrimination across all studies.

Table 2 describes the pooled prevalence estimates for IHD. The pooled prevalence for any form IHD during residency training was 64.1% (52 studies; 63,378 residents; 95% CI: 51.0-77.1%; *I^2^* = 99.96%, Figure 2).Residents reported verbal harassment (cited by 61.5% of respondents) as the most common form of IHD, followed by physical (30.0%), sexual (28.0%), work as punishment (26.9%), academic (26.5%), loss of privileges or opportunities (9.5%), and retaliation (4.8%). Heterogeneity was significant across these studies.

**Table 2:** Prevalence of Intimidation, Harassment, and Discrimination (IHD) among residents

	*No*. *of* *Studies*	*Sample* *Size* *(n)*	*Prevalence*	*95%* *CI*	*I*^2^
**Types**					
**Overall**	52	22,549	64.1%	51.0-77.1	99.9
**Verbal**	34	15,987	61.5%	47.0-75.9	99.9
**Sexual**	28	2,927	28.0%	20.6-35.4	99.8
**Physical**	39	4,621	30.0%	22.6-37.5	99.7
**Work as punishment**	11	2,302	26.9%	6.9-46.9	99.9
**Academic**	9	896	26.5%	15.3-37.7	98.9
**Loss of privileges**	6	285	9.5%	6.2-12.0	88.2
**Retaliation**	4	78	4.8%	1.9-7.6	84.0
**Other types**	8	1,243	23.5%	11.5-35.5	99.6
**Repeated**	7	1,025	52.1%	32.3-72.0	98.9
**Sources**					
**Staff Physicians**	28	10,371	39.0%	30/5-47.4	99.8
**Nurses**	21	6,471	47.8%	26.0-69.6	100.0
**Residents**	24	6,002	35.8%	8.9-62.6	100.0
**Medical Students**	5	506	10.6%	3.2-18.0	98.7
**Patients**	22	8,739	41.7%	34.3-49.2	99.9
**Families/relatives**	4	1,072	50.7%	28.6-72.8	99.1
**Other staff**	13	1,155	9.5%	7.1-12.0	98.0
**Basis/Risk Factors**					
**Gender**	9	1860	41.7	22.7-60.6	99.5
**Training status**	1	599	55.5	52.5-58.5	N/A
**Sexual orientation**	4	310	2.5	0.5-4.6	84.4
**Ethnicity**	8	1955	20.6	13.2-28.1	98.9
**Culture**	4	156	9.9	5.1-14.6	91.8
**Language**	3	31	2.3	0.6-4.0	75.2
**Another basis**	5	561	26.2	4.7-47.7	99.5

CI = confidence interval

I^2^ = measure of statistical heterogeneity (higher indicates greater heterogeneity)

Other types = any other form of IHD reported by residents that was not consistent with one of the other categories (e.g., economic abuse)

Other staff = any other source of IHD from employees that were not staff physicians or nurses (such as administrators, housekeeping, or clerical staff)

### Post hoc subgroup analyses

Post hoc subgroup analyses were conducted to identify trends in the prevalence of IHD. There were no statistically significant differences in the prevalence of IHD in studies using national, local, or regional samples of residents, or in studies that provided definitions of IHD (relative to those that did not). However, IHD was more prevalent among female residents compared to male residents (*p*< 0.05) and amongst residents of visible minorities (including Asian, Middle Eastern, or Black residents) relative to Caucasian residents (*p*< 0.05). However, neither the proportion of female respondents nor the response rate to the survey were significantly associated with the overall prevalence of IHD in meta-regression.

### IHD among psychiatry residents

Table 3 outlines the IHD characteristics among psychiatry residents. The overall response rate among psychiatry residents was 41.0% (15 studies; N= 2,377/5,794, n = 15 studies), while the average response rate per study was 68%. The pooled prevalence of IHD among psychiatry residents was 59.5% (95% CI: 0.393-0.796, *I^2^* = 99.7%). The most common types of IHD reported by psychiatry residents was verbal (66.4%; n = five studies), physical (46.0%; n = 12 studies), and sexual (39.5%; n = 4 studies). The most common sources of IHD reported by psychiatry residents were patients (57.1%; n = 12 studies) followed by staff physicians (29.5%; n = 2 studies).

**Table 3:** Prevalence of Intimidation, Harassment, and Discrimination (IHD) among psychiatry residents

	*No*. *of* *Studies*	*Sample* *Size* *(n)*	*Prevalence*	*95%* *CI*	*I*^2^
**Types**					
**Overall**	15	2377	59.5%	39.3-79.6	99.7
**Verbal**	5	701	66.4%	56.9-75.8	88.8
**Sexual**	4	225	39.5%	6.7-72.3	99.4
**Physical**	12	692	46.0%	20.8-71.3	99.5
**Repeated**	3	305	41.3%	-1.0-83.7	99.7
**Sources**					
**Staff Physician**	1	77	8.9%	4.5-13.4	23.2
**Patients**	1	1167	21.7%	15.2-28.1	98.2

CI = confidence interval

I^2^ = measure of statistical heterogeneity (higher indicates greater heterogeneity)

### Reporting of IHD

The pooled prevalenceofreporting awareness of how to report IHD incidents was 41.0% (N = 2311/4416; n = 7 studies; 95% CI = 20.8-61.1%; *I^2^* = 99.548%), while the pooled rate of reporting of IHD incidents was only 26.9% (N = 2080/9155; n = 9 studies; 95% CI = 16.2-35.2%; *I^2^* = 99.747%). The top barriers to reporting were fear of retaliation, that residentsbelieved they could handle the incident on their own, that they believed the IHD incident was not significant enough to warrant reporting, that reporting would not improve the situation, or a lack of awareness of reporting infrastructure.

### Impact of IHD

Twenty-four studies (46%) reported the impact of IHD on residents, including self-reported psychological sequelae (n = 13), positive screening for common mental health disorders (n = 4), measures of overall life satisfaction (n = 4), self-report of subjective feelings of safety at work (n = 2), and overall disruption in residents’ ability to work (n = 2). The majority of psychological sequelae of IHD reported by residents was negative and harmful, including perceived hostility at work, increasing emotionality, anger, frustration, burnout, diminished resilience, increased substance abuse (as a maladaptive coping mechanism for stress), anxiety, depression, fear, and feelings of inadequacy. 75.4% (N = 2142/2789; n = 4 studies; 95% CI = 71.6-79.2%; *I^2^* = 66.3%) reported their general quality of life to be “good” or “very good” on a 5-point Likert scale, suggesting that respondents were generally resilient to the effects of IHD. However, 45.0% (N = 949/2789; n = 4 studies; 95% CI = 5.9-84.2%; *I^2^* = 99.8%) indicated their mental health to be “Fair” or “Poor” on a 5-point Likert scale.

### Proposed resources and solutions to IHD

Most studies reported proposed resources and potential solutions to IHD. These included IHD education and training (90%; n = 47 studies); anti-IHD policies, infrastructure, or administrative changes (83%; n = 43 studies); access to supports, such as friends, family, and program directors (35%; n = 18 studies); participation in wellness activities (23%; n = 12 studies); access to an ombudsperson (13%; n = 7); support and counselling from their family physician (13%; n = 7 studies); access to psychological therapy or a psychiatrist (15%; n = 8 studies); access to career support or advice (12%; n = 6 studies); and access to financial support or advice (12%; n = 6 studies).

### Sensitivity analysis

Heterogeneity was significant across these studies; however, the prevalence estimates across meta-analyses were robust to the leave-out-one test of significance.

## Discussion

### Summary

The present studyprovides an updated systematic review and meta-analysis on the prevalence of IHD among resident physicians. To the best of our knowledge, this review is the largest to specifically focuses on IHD among resident physicians, including 63,378 trainees in total.

The pooled prevalence of IHD was 64.1% (95% CI, 51.0-77.1). Verbal, physical, and sexual IHD were the most commonly reported forms of IHD. Training status (55.5%), gender (41.7%), and ethnicity (20.6%) were the most commonly reportedperceived risk factors for IHD. The most common sources of IHD were relatives/friends of patients, nurses, and patients themselves (reported by 50.7%, 47.8, and 41.7%, respectively).

Residents described several negativeeffectsofIHD—including poorer overall mental health, lower quality of life, and decreased satisfaction with work. Residents frequently screened positive for multiple psychiatric disorders, including depression, anxiety, substance abuse, and suicidality. However, residents also proposed several potential resources and solutions for addressing IHD, including education about IHD, decreasing barriers to reporting IHD, and increasing access to supports.

### IHD among psychiatry residents

Although the overall prevalence of IHD among psychiatry residents (59.5%) was similar to the overall mean (64.1%), the prevalence of sexual IHD (39.5%) and physical IHD (46.0%) were significantly higher (*p*< 0.05). Although the latter may be due to the overrepresentation of violence-focused studies among psychiatry trainees, the former may relate to the unique characteristics of the psychiatric discipline, which we attempt to explain here.

Several studies have shown that the ways in which residents and medical students experience psychiatry training is different than in other specialties, which may extend to experiences of IHD during training.^68^^-^^70^ However, the available literature exploring the nature of the learning and training environment in psychiatry is controversial, with one study suggesting that perceptions and personal experiences of IHDwithin the psychiatric learning environment are low.^71^Previous studies have consistently demonstrated elevated rates of IHD among psychiatry trainees relative to other trainee groups.^36^^,^^72^^-^^74^ This is likely related to unique power differentials,^24^ which may be driven by prejudicial views toward the discipline of psychiatry that disproportionately sensitize trainees to IHD.^75^ Factors related to compassion fatigue and burnout may also be more common among psychiatry trainees given the empathic demands of their work and vicarious exposure to IHD.^69^ Psychiatrists are also paid the least among medical specialties—a systematic factor that is outside of the immediate control of most physicians.^76^^-^^78^ As IHD is considered a major risk factor for burnout, with a 2016 national survey of Canadian psychiatry residents finding that 21% reported symptoms of burnout, IHD encounters appear to play a critical role in psychiatry resident experiences of burnout.^79^

### Heterogeneity of results

A major source of heterogeneity in our meta-analysis arose from variations in the type of IHD terminology—including specific definitions or examples—used across studies. Although the majority of studies (90.4%) provided definitions, five^80^^-^^84^ intentionally did not in order to limit the restraint on respondents. However, the presence of IHD definitions was not associated with IHD prevalence. Among thestudies incorporating IHD definitions, the majority used terminology that were based on how the respondent perceived IHD, rather than objective criteria. For example, most studies included “uncomfortable propositions of a sexual nature” and “unwanted sexual banter” under “sexual harassment”, so respondents that perceived sexually themed interactions as unwanted would respond positively to such questions.

### Significance of findings

This review indicates that IHD is a highly prevalent phenomenon among residents of most specialties. Specific risk factors that may increase vulnerability to IHD among resident physicians are not consistently defined in the academic literature. However, this review found that female residents and those belonging to visible minorities were at greater risk.

While the culture of medicine and residents’ lack of control over their schedules have been previously cited as the biggest barriers to seeking mental health care,^85^it remains unclear if these factors extend to vulnerability to IHD. However, studies of IHD in otherprofessions may be relevant in defining the vulnerability profile of IHD within medicine. Among army soldiers, personality traits, such as “negative femininity”—which reflects extreme passivity—and “negative masculinity”—which includes antisocial characteristics—were both positively correlated with unwanted sexual experiences among male and female soldiers.^86^Similarly, students who underestimated their own likelihood of being sexually assaulted were at greater risk compared to their peers.^87^

Still, vulnerable targets may be less obvious in the medical workplace. Medical students, residents, fellows, postdoctoral fellows and junior faculty are dependent on their superiors for recommendation letters, evaluations, opportunities, mentorship, among other reasons.^23^ Residents who have experienced IHD during their training frequently report disappointment with the effectiveness of existing anti-IHD infrastructure,^67^ fear of reprisal and retaliation, and identify numerous barriers to reporting IHD.^33^

### Comparison to other reviews

The prevalence estimates of the presentmeta-analysis are consistent with prior IHD reviews.^36^^,^^88^^,^^89^Fnais and colleagues found that 63.4% of residentshad experienced at least one form IHD during their training.^36^ Karim and Duchchererfound that the prevalence of IHD in residency varied between 45% and 93%.^6^ Huang and colleagues found that discrimination occurred in 22.4% of surgeons and surgery trainees [95% CI = 14.0–33.9%]; however, as this was a pooled estimate of medical students, residents, fellows, and staff, it is not directly comparable to our study’s estimate, which is exclusive to residents. Interestingly, one of the component studies in the Huang meta-analysis found that among surgical residents only, rates of bullying varied from 11.5-82.5%.

Although the general findings of our study are consistent with IHD literature,^14^^,^^18^^,^^22^^-^^24^ IHD prevalence appears to be higher among residents than staff physicians. However, among staff physicians, IHD prevalence is highly variable. For example, in a recent survey of radiologists, only 10% of respondents reported sexual harassment,^90^ while a recent survey of all Australasian surgeons found that 49.2% reported experiencing bullying, discrimination and sexual harassment behaviours.^91^

Our study’s findings are also consistent with reports of high prevalence of IHD in non-medical institutions of higher learning. For example, nearly 30% of college students have previously reported sexual harassment during their education.^22^ This finding lends support to the trans-institutional nature of academic IHD.^92^^-^^95^

## Limitations

As our study is a meta-analysis of cross-sectional surveys, issues of selection and information bias are especially relevant. For example, given that the overall response rate was only about 50%, there is a high possibility of selection bias if the non-responders were significantly different than the responders with respect to their experience of IHD. Furthermore, as surveys were based on self-report and required the respondent to recall the duration of their residency experience, this also introduces a high chance of recall bias.

Most surveys included in the meta-analysis provideddefinitions of IHD. While this may improve the consistency of reporting within a study, definitions were inconsistently used across studies, and may have restricted respondents in how they interpreted their personal experiences of IHD; however, we did not identify a significant difference in IHD prevalence between studies that used definitions and those that did not. Given the subjective nature of IHD, additional factors, such as social desirability, confidentiality, and fear of reprisal, may have influenced the ability of respondents to truthfully complete the surveys.

Although we included all types and kinds of IHD, there were a limited number of studies reporting certainrisk factors for IHD, such as training status, which was only reported by one study. However, this one risk factor was found to be highly cited (by 55.5% of respondents), suggesting training status is a significant risk factor for IHD.

Despite inclusion of a grey literature source, the majority of eligible studies identified by this review were published, English-language studies of resident physicians based in the United States, Canada, and the United Kingdom. Therefore, the generalizability of our findings may be limited to these populations.

While all postgraduate year residents were included, 71% were in their first two years, and only a minority of respondents from postgraduate year three or onwards were represented. This is potentially significant as the experiences of more senior residents may be different from more junior trainees.

As our meta-analysis synthesized surveys across decades and across countries, this may have introduced additional heterogeneity in the results. Literature on the reporting of various forms of IHD has suggested that rates of reporting of IHD are on the rise.^10^^,^^96^^-^^98^Demographic differences in resident populations, such as greater representation of women^99^^,^^100^ and minority groups,^101^^,^^102^ may have contributed to increasing rates of IHD reporting in more recent studies compared to older ones.^103^

### Strengths

Our study has a number of strengths. First, our meta-analysis is up to date, including several recent studies from 2018 and onwards. Second, our study is comprehensive, synthesizing 52 studies, 63,378 respondents, and multiple medical specialties. Third, our inclusion of all types, sources, and risk factors for IHD enables a rich and thorough discussion about trends in IHD among residents. Fourth, our focus on diverse secondary outcomes including reporting measures, impact measures, and potential solutions to IHD, allows our study to unique contribute to the available literature on IHD among resident physicians, and IHD in medicine.

### Future directions

While IHD continues to be a highly prevalent and serious issue for resident physicians, there is hope that a future without IHD in medicine is possible.^104^^-^^107^ Future research should explore the efficacy of anti-IHD interventions, such as education or policy change, on the overall prevalence of IHD.

### Conclusion

Our study achieved its proposed aim of establishing the prevalence of IHD among resident physicians, IHD risk factors, and potential sources and solutions to IHD. Despite growing recognition of IHD in medical education, the responses of the medical and medical education systems and organizations to IHD has been inadequate. Given the high rates and severe consequences of IHD, it is disappointing that the situation remains unchanged after many years. However, we end our study with a call to action—that future researchers identify effective intervention and prevention strategies to reduce IHD and its sequelae

## Appendices

### Appendix A Types, definitions and examples of Intimidation, Harassment, and Discrimination (IHD) terminology

**Table:**

Type of IHD	Examples
**Verbal**	Shouting or raising one’s voice Ridicule Constant interruption and refusing to listen Singling someone out for grilling or interrogation
**Sexual**	Disrespectful jokes or banter about sex Comments about someone’s physical appearance or sexual attractiveness
**Physical**	Unwelcome physical contact Physical intimidation/harassment, e.g. pushing, punching, slapping, threatening gestures, or throwing objects at an individual
**Work as Punishment**	Unjust assignment of duties Overloading someone with work Education/service imbalance e.g. contractual infractions, inadequate supervision, excessive service load or service assignment without educational merit
**Academic**	Being asked to carry out some personal services unrelated to patient care or educational activities Questions/queries were intentionally not answered You were threatened with failure or giving poor evaluations for reasons unrelated to your academic performance
**Loss of Privileges or Opportunities**	Privileges/opportunities taken away unfairly or in ways that are not related to resident’s performance
**Retaliation/Recrimination**	Reprisal or threat of reprisal for negative feedback of staff, program or service, including the lodging of a complaint or grievance
**Other**	Economic abuse
**Repeated**	Situations where residents perceive more than one instance of intimidation, harassment or discrimination
**Risk Factors/Basis for IHD**	Examples
**Gender**	Comments about one’s gender identity or gender expression Sexist teaching materials Punitive measures and favoritism based on gender
**Training Status**	Intimidation, harassment or discrimination on the basis of the resident’s rank (e.g., postgraduate year 1 versus a postgraduate year 5), relying on intrinsic hierarchical systems
**Sexual Orientation**	Homophobic remarks Assumptions on the basis of the residents’ perceived sexual orientation
**Ethnicity**	Racial epithets or slurs Negative stereotypes about a particular ethnic group
**Culture**	Disparagement of someone’s cultural or religious devotions
**Language**	Rude or disparaging remarks on the basis of someone’s first language or perceived difficulty with the native language
**Other**	Physical appearance, location of training, region of training

### Appendix B Literature search strategy

**PubMed: inception to May 28, 2019**

**Table:**

**1**	**((((resident) OR trainees) OR intern)) AND ((((intimidation) OR harassment) OR discrimination)))**	**678**

**OVID MEDLINE: inception to May 28, 2019**

**Table:**

1	exp “Internship and Residency”/	45,936
2	exp Sexual Harassment/ or harassment.mp. or exp Bullying/	6,873
3	violence.mp. orexp Violence/	106,650
4	intimidation.mp.	510
5	discrimination.mp. orexp “Discrimination (Psychology)”/	138,628
6	2 or 3 or 4 or 5	249,378
**7**	**1 and 6**	**490**

**OVID EMBASE: inception to May 28, 2019**

**Table:**

1	resident physician.mp. or exp resident/	35,434
2	exp bullying/ or intimidation.mp. or exp violence/	145,170
3	exp non-sexual harassment/ or harassment.mp. or exp harassment/ or exp sexual harassment/	4,786
4	discrimination.mp.	181,619
5	2 or 3 or 4	326,212
**6**	**1 and 5**	**512**

**OVID PsycINFO: inception to May 28, 2019**

**Table:**

1	Exp Violence/ or exp Bullying/ or exp Harassment/ or exp Threat/ or intimidation.mp	93,572
2	exp DISCRIMINATION/ or discrimination.mp.	98,304
3	exp Medical Residency/ or resident physician.mp.	4,394
4	1 or 2	190,243
**5**	**3 and 4**	**78**

**Allied and Complementary Medicine (AMED): inception to May 28, 2019**

**Table:**

1	resident physician.mp.	9
2	resident.mp.	533
3	medical resident.mp.	5
4	Physicians/ or physicians.mp.	4,837
5	1 or 2 or 3 or 4	5,293
6	intimidation.mp.	15
7	Sexual harassment/ or harassment.mp.	75
8	Discrimination/ or discrimination.mp.	996
9	Violence/ or violence.mp.	733
10	bullying.mp.	98
11	6 or 7 or 8 or 9 or 10	1,852
**12**	**5 and 11**	**24**

**Cochrane Library: inception to May 28, 2019**

**Table:**

1	resident physician.mp. [mp=title, short title, abstract, full text, keywords, caption text]	6
2	resident.mp. [mp=title, short title, abstract, full text, keywords, caption text]	206
3	physician.mp. [mp=title, short title, abstract, full text, keywords, caption text]	1,779
4	intimidation.mp. [mp=title, short title, abstract, full text, keywords, caption text]	7
5	harassment.mp. [mp=title, short title, abstract, full text, keywords, caption text]	11
6	discrimination.mp. [mp=title, short title, abstract, full text, keywords, caption text]	188
7	bullying.mp. [mp=title, short title, abstract, full text, keywords, caption text]	28
8	violence.mp. [mp=title, short title, abstract, full text, keywords, caption text]	248
9	1 or 2 or 3	1,918
10	4 or 5 or 6 or 7 or 8	426
**11**	**9 and 10**	**87**

**CINAHL: inception to May 28, 2019**

**Table:**

1	(MH “Interns and Residents”) OR “resident physician” OR (MH “Physicians”)	59,139
2	“intimidation”	332
3	(MH “Sexual Harassment”) OR (MH “Cyberbullying”) OR (MH “Bullying”) OR “harassment”	9523
4	(MH “Discrimination”) OR “discrimination” OR (MH “Sexism”) OR (MH “Racism”) OR (MH “Ageism”) OR (MH “Discrimination, Employment”)	35,120
5	2 or 3 or 4	44,163
**6**	**1 and 5**	**444**

**ProQuest: inception to May 29, 2019**

**Table:**

**1**	**“resident physician” AND (“intimidation” OR “harassment” OR “discrimination”)**	**928**

### Appendix C Data coding guide

**Study characteristics**

- Author(s)
- Year
- Country
- Study Design & Format
- Surveying Institution (university, organization)
- Surveyed Distribution
- Total (n)
- Responder (n)
- Response rate (%)
- Were definitions of IHD included? (yes/no)
- What types of IHD were reported? (types)

**Resident characteristics**

- Specialty (descriptor)
- Male responders (n)
- Female responders (n)
- Domestic medical graduates (n)
- PGY1 (n)
- PGY2 (n)
- PGY3 (n)
- PGY4 (n)
- PGY5+ (n)
- Age (mean)
- Age (SD)

**Baseline measures**

- Were work hours measured/reported? (yes/no)
- Was a measurement/metric of stress/distress reported? (yes/no)
- Top contributing factors for stress (descriptor)

**Primary outcomes: prevalence of IHD by types**

- Overall IHD (n)
- Verbal IHD (n)
- Sexual IHD (n)
- Physical IHD (n)
- Work as punishment IHD (n)
- Academic IHD (n)
- Loss of Privileges/Opportunities IHD (n)
- Retaliation/Recrimination IHD (n)
- Other IHD (n)
- Repeat IHD (n)

**Secondary outcomes: Sources of IHD**

- Staff Physicians (n)
- Nurses (n)
- Residents (n)
- Medical Students (n)
- Patients (n)
- Families (n)
- Other Staff (n)

**Secondary outcomes: basis/risk factors for IHD**

- Gender (n)
- Training Status (n)
- Sexual Orientation (n)
- Ethnicity (n)
- Culture (n)
- Language (n)
- Other (n)

**Secondary outcomes: Reporting IHD**

- Awareness of Reporting Infrastructure (n)
- Number who Actually Reported IHD (n)
- Top Barriers to Reporting

**Secondary: Impact on resident health**

- What impact metrics were reported?
- General Satisfaction/Quality of Life Good (n)
- Self-Rated MH Fair/Poor (n)
- Was MH Screening Done? (yes/no)

**Secondary: Resources identified by residents/proposed by study**

- IHD-specific Education/Training (yes/no)
- IHD-specific Policies/Infrastructure/Administration Changes (yes/no)
- Access to Supports (yes/no)
- Wellness (yes/no)
- Access to an Ombudsperson (yes/no)
- Access to a Family Physician/GP (yes/no)
- Access to Psychology/Psychiatry (yes/no)
- Career Advice (yes/no)
- Financial Advice (yes/no)

### Appendix D Raw data

Available from authors upon request.

### Appendix E Risk of bias tool definitions

External Validity

1. Was the study’s target population a close representation of the national population in relation to relevant variables?
2. Was the sampling frame a true or close representation of the target population?
3. Was some form of random selection used to select the sample, OR was a census undertaken?
4. Was the likelihood of nonresponse bias minimal?

Internal Validity

1. Were data collected directly from the subjects (as opposed to a proxy)?
2. Was an acceptable case definition used in the study?
3. Was the study instrument that measured the parameter of interest shown to have validity and reliability?
4. Was the same mode of data collection used for all subjects?
5. Was the length of the shortest prevalence period for the parameter of interest appropriate?
6. Were the numerator(s) and denominator(s) for the parameter of interest appropriate?

Overall

1. Summary item on the overall risk of study bias.

### Appendix F Risk of bias assessment

**Table:**

Study	Population	Sample	Randomization	Response	Collection	Definition	Instrument	Consistency	Duration	Parameter	Total	Rating	
**Acik et al. 2008**	0	0	1	0	0	0	1	0	0	0	2		Low
**Al-Shafaee et al. 2013**	0	0	1	0	0	0	1	0	0	0	2		Low
**Alimohammadi et al. 2013**	0	0	1	0	0	0	1	0	0	0	2		Low
**Baldwin et al. 1996**	0	0	0	0	0	0	1	0	0	0	1		Low
**Baldwin et al. 1997**	0	0	1	0	0	0	1	0	0	0	2		Low
**Baldwin et al. 1994**	0	0	1	0	0	0	1	0	0	0	2		Low
**Barlow & Rizzo 1997**	0	0	0	1	0	0	1	0	0	0	2		Low
**Behnam et al. 2011**	0	0	0	0	0	1	1	0	0	0	2		Low
**Black et al. 1994**	1	0	1	0	0	1	1	0	0	0	4		Mod
**Carr et al. 1991**	0	0	1	0	0	0	0	0	0	0	1		Low
**Chadaga et al. 2016**	0	0	0	0	0	0	1	0	0	0	1		Low
**Chaimowitz&Moscovitch 1991**	0	0	1	0	0	0	0	0	0	0	1		Low
**Cohen & Patten 2005**	1	0	1	0	0	1	0	0	0	0	3		Mod
**Cohen et al. 2008**	0	0	1	1	0	1	0	0	0	0	3		Mod
**Cook et al. 1996**	1	0	1	0	0	0	0	0	0	0	2		Low
**Crutcher et al. 2011**	1	0	1	0	0	0	0	1	0	0	3		Mod
**Daugherty et al. 1998**	0	0	0	0	0	0	1	0	0	0	1		Low
**Deringer &Caligor 2014**	1	0	1	0	0	1	1	0	0	0	4		Mod
**Dvir et al. 2001**	0	0	0	1	0	0	1	0	0	0	2		Low
**Fink et al. 1991**	1	0	1	1	0	0	1	0	0	0	4		Mod
**Finucane & O'Dowd 2005**	0	0	1	0	0	0	1	0	0	0	2		Low
**Fnais et al. 2003**	0	0	1	0	0	0	1	0	0	0	2		Low
**Gray 1989**	0	0	1	0	0	0	1	0	0	0	2		Low
**Hoosen & Callaghan 2004**	0	0	1	0	0	0	1	0	0	0	2		Low
**Hostiuc et al. 2014**	1	0	1	0	0	0	1	0	0	0	3		Mod
**Judy &Veselik 2009**	0	0	1	1	0	0	1	0	0	0	3		Mod
**Keeley et al. 2005**	0	0	1	0	0	0	1	0	0	0	2		Low
**Komaromy et al. 1993**	1	0	1	0	0	0	1	0	0	0	3		Mod
**Kozlowska et al. 1997**	0	0	1	0	0	0	1	0	0	0	2		Low
**Li et al. 2010**	1	0	1	0	0	0	1	0	0	0	3		Mod
**Mackin 2001**	0	0	1	0	0	0	1	0	0	0	2		Low
**McNamara et al. 1995**	0	0	0	0	0	0	1	0	0	0	1		Low
**Milstein 1987**	1	0	1	0	0	0	1	0	0	0	3		Mod
**Milstein et al. 1987**	1	0	1	0	0	0	1	0	0	0	3		Mod
**Morgan & Porter 1999**	0	0	0	0	0	0	1	0	0	0	1		Low
**Nagata-Kobayashi et al. 2009**	0	0	1	0	0	0	0	0	0	0	1		Low
**Ogunsemi et al. 2010**	1	0	1	0	0	0	0	0	0	0	2		Low
**Paice& Smith 2009**	0	0	1	0	0	0	1	0	0	0	2		Low
**Paice et al. 2004**	0	0	1	0	0	0	0	0	0	0	1		Low
**Pieters et al. 2005**	0	0	1	0	0	0	0	0	0	0	1		Low
**Quine 2002**	0	0	0	0	0	0	1	0	0	0	1		Low
**RDoC 2011**	0	0	1	1	0	0	0	0	0	0	2		Low
**RDoC 2012**	0	0	1	1	0	0	0	0	0	0	2		Low
**RDoC 2013**	0	0	1	1	0	0	0	0	0	0	2		Low
**RDoC 2018**	0	0	1	1	0	0	0	0	0	0	2		Low
**Recupero et al. 2005**	1	0	1	0	0	0	1	0	0	0	3		Mod
**Ruben et al. 1989**	1	0	1	0	0	0	1	0	0	0	3		Mod
**Schnapp et al. 2016**	1	0	1	0	0	0	0	0	0	0	2		Low
**Schwartz & Park 1999**	0	0	0	1	0	0	0	0	0	0	1		Low
**Vaninevald et al. 1996**	0	0	1	0	0	0	0	1	0	0	2		Low
**Vukovic et al. 1996**	0	0	1	0	0	0	0	0	0	0	1		Low
**Walter et al. 2003**	0	0	1	1	0	0	1	0	0	0	3		Mod
